# Prioritization of candidate genes in “*QTL-hotspot*” region for drought tolerance in chickpea (*Cicer arietinum* L.)

**DOI:** 10.1038/srep15296

**Published:** 2015-10-19

**Authors:** Sandip M Kale, Deepa Jaganathan, Pradeep Ruperao, Charles Chen, Ramu Punna, Himabindu Kudapa, Mahendar Thudi, Manish Roorkiwal, Mohan AVSK Katta, Dadakhalandar Doddamani, Vanika Garg, P B Kavi Kishor, Pooran M Gaur, Henry T Nguyen, Jacqueline Batley, David Edwards, Tim Sutton, Rajeev K Varshney

**Affiliations:** 1 International Crops Research Institute for the Semi-Arid Tropics (ICRISAT), Center of Excellence in Genomics (CEG), Hyderabad, 502324, India; 2 Osmania University, Department of Genetics, Hyderabad, 500007, India; 3 The University of Western Australia, School of Plant Biology and the Institute of Agriculture, Crawley, 6009, Australia; 4 University of Queensland, School of Agriculture and Food Science, Queensland, 4072, Australia; 5 Oklahoma State University, Department of Biochemistry and Molecular Biology, Stillwater, 74074, USA; 6 Cornell University, Biotechnology Building, Ithaca, 14853, USA; 7 University of Missouri, National Center for Soybean Biotechnology and Division of Plant Sciences, Columbia, 65211, USA; 8 South Australian Research and Development Institute, Adelaide, 5001, Australia; 9 University of Adelaide, Australia and School of Agriculture, Adelaide, 5064, Australia

## Abstract

A combination of two approaches, namely QTL analysis and gene enrichment analysis were used to identify candidate genes in the “*QTL-hotspot*” region for drought tolerance present on the Ca4 pseudomolecule in chickpea. In the first approach, a high-density bin map was developed using 53,223 single nucleotide polymorphisms (SNPs) identified in the recombinant inbred line (RIL) population of ICC 4958 (drought tolerant) and ICC 1882 (drought sensitive) cross. QTL analysis using recombination bins as markers along with the phenotyping data for 17 drought tolerance related traits obtained over 1–5 seasons and 1–5 locations split the “*QTL-hotspot*” region into two subregions namely “*QTL-hotspot_a*” (15 genes) and “*QTL-hotspot_b*” (11 genes). In the second approach, gene enrichment analysis using significant marker trait associations based on SNPs from the Ca4 pseudomolecule with the above mentioned phenotyping data, and the candidate genes from the refined “*QTL-hotspot*” region showed enrichment for 23 genes. Twelve genes were found common in both approaches. Functional validation using quantitative real-time PCR (qRT-PCR) indicated four promising candidate genes having functional implications on the effect of “*QTL-hotspot*” for drought tolerance in chickpea.

Chickpea (*Cicer arietinum* L.) crop is a rich source of protein (20–30%), carbohydrates (~40%) and minerals and thus serves as an important source of nutrients in vegetarian diets, especially in developing countries^1^. However, its productivity is severely affected by various abiotic stresses such as drought, heat and salinity. Among these, terminal drought is one of the most serious production constraints, often leading to 40–50% annual yield loss, as the crop is largely cultivated on residual soil moisture in the arid and semi-arid regions of South Asia and sub-Saharan Africa^2^. Additionally, global warming and predicted future climatic change primarily affecting precipitation, temperature and evapotranspiration will further increase the drought issue. Therefore, enhancing drought tolerance in chickpea is required to enhance crop productivity. To supplement conventional breeding, some efforts such as QTL mapping^3^,^4^,^5^, candidate gene based allele diversity analysis^6^ and genome wide association study (GWAS)^7^ have been made to identify QTL/genes for drought tolerance related traits in chickpea. In our earlier study, by using phenotyping data for 20 drought tolerance related traits collected in 1–7 seasons at 1–5 locations in India and genotyping data for 241 simple sequence repeat (SSR) loci on one intra-specific population (ICC 4958 × ICC 1882), we identified a “*QTL-hotspot*” region harbouring 12 QTLs for 12 drought tolerance related traits explaining up to 58.20% phenotypic variation^5^. Introgression of this “*QTL-hotspot*” region in JG 11, an elite variety, has improved root traits and drought tolerance^8^. However, this region was estimated to be 29 cM on the genetic map and 7.74 Mb on the physical map^9^. Subsequently, by using a genotyping-by-sequencing (GBS) approach, we refined this region to 14 cM, equivalent to ca. 3 Mb^10^. Therefore, for identification of candidate genes associated with drought tolerance as well as development of closely linked markers for breeding, there is a need for fine mapping the “*QTL-hotspot*” region.

Recent advances in sequencing technology have provided a cost effective way to develop several thousand SNPs in a limited period of time in large mapping populations, by sequence-based genotyping^11^,^12^. One such technique, GBS^13^, offers simultaneous SNP detection and scoring and therefore is now being used in several crops for diversity assessment, trait mapping, GWAS and genomic selection^14^,^15^. This approach has already been used by us and as mentioned above, we refined the “*QTL-hotspot*” region to ca. 3 Mb^10^. The GBS approach, however, suffers from the limitation of missing regions of the genome and bias based on methylation and restriction site abundance^16^. An alternate approach to overcome missing data issues is whole genome re-sequencing (WGRS) wherein samples can be sequenced at greater depth thereby reducing the missing data issue. To save the cost of sequencing, WGRS can be done at a lower depth and in that scenario the approach is referred to as skim sequencing^17^. This approach is very useful to identify sequence variants/SNPs in species where the reference genome sequence is available. However, the SNPs identified using NGS technologies cannot be directly used for QTL studies as: i) Next generation sequencing (NGS) technologies are prone to small but unrecoverable sequencing errors and therefore an individual SNP site cannot be used as a reliable marker for genotyping, ii) it is very difficult to score all SNP sites in an entire recombinant population, and iii) limitations of QTL analysis software to handle such a huge dataset. To address these issues, a parent dependant sliding window approach was used to identify true recombination breakpoints and to construct a recombination bin map using SNP data of an entire recombinant population in rice^18^. Using a bin mapping approach, a QTL for plant height was reported^18^ within the region that contained the semi-dwarf gene, *sd1*, responsible for the rice “green revolution”^19^. By using the WGRS bin mapping approach, candidate gene(s) were identified for root-knot nematode resistance^20^ and salt tolerance^21^ in soybean and for grain width in rice^22^.

With an objective of fine mapping the “*QTL-hotspot*” region and to identify candidate genes, we sequenced the intraspecific RIL mapping population derived from ICC 4958 (drought tolerant genotype) and ICC 1882 (drought sensitive genotype) cross and constructed a bin map. By using a combination of bin mapping based QTL analysis and *a priori* gene enrichment analysis, a set of candidate genes were prioritized. Functional validation of these genes has provided the most promising candidate genes that are involved in regulation of drought tolerance in chickpea.

## Results

### High-density bin mapping

#### Skim sequencing and SNP calling

**Figure 1:**
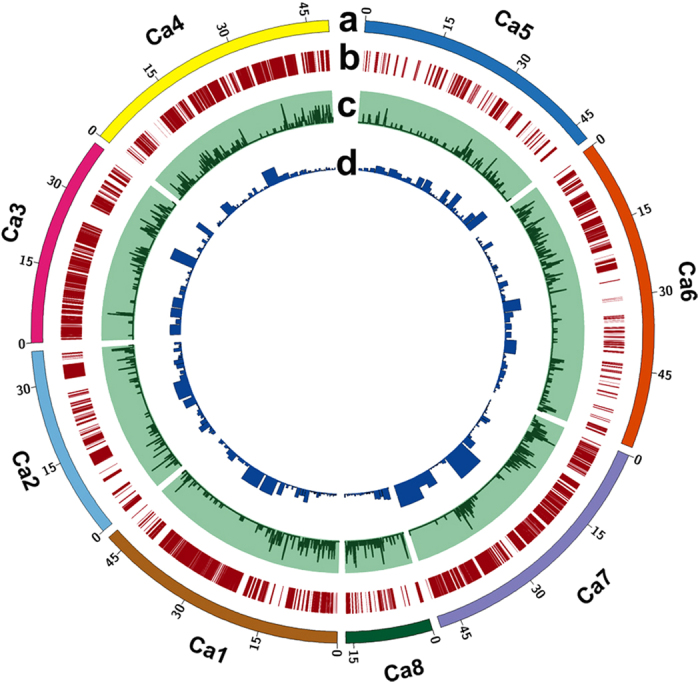
Genome-wide distribution of SNPs and recombination bins in chickpea. (**a**) Chickpea pseudomolecules, labelled as Ca1 to Ca8 and each pseudomolecule is shown in a different colour. The numbers on arches represent the scale for the size of pseudomolecules in Mb; (**b**) Distribution of 53,223 SNPs on eight chickpea pseudomolecules. Each SNP is represented as a single vertical line. The highest number of SNPs (18,989) were identified on Ca4 while the lowest number (954) of SNPs were identified on Ca5. Aggregation of vertical lines indicates SNP dense regions, while SNP sparse regions are depicted by blank spaces. Pseudomolecules Ca1, Ca3, Ca4 and Ca7 were found to contain SNP dense regions, whereas SNP sparse regions were observed on Ca2, Ca5, Ca6 and Ca8 pseudomolecules; (**c**) Distribution of 1,610 recombination bins on chickpea pseudomolecules. The number of recombination bins within 100 Kb intervals were calculated and plotted as a smooth line curve. The height of the line indicates the number of bins within the respective 100 Kb interval. A flat line corresponds to no or limited recombination regions; (**d**) Distribution of genes among recombination bins. The number of genes located within each recombination bin were identified by comparing the coordinates of the respective bin with chickpea gene models. The width of the column is proportional to the recombination bin interval while column height is proportional to the number of genes within that interval.

The RIL population (ICC 4958 × ICC 1882) was genotyped using a skim sequencing approach. In the first instance, a total of 5.90 Gb and 5.94 Gb data were generated for ICC 4958 and ICC 1882 respectively, which represents an estimated coverage of 7.90X and 8.03X. SGSautoSNP identified 84,963 SNPs, out of which 64,581 were distributed over the 8 pseudomolecules and 20,382 were located on unplaced contigs. Subsequently, a total of 497.60 Gb of Illumina paired read sequence data were generated for 232 RIL samples. The data obtained ranges from 0.21X to 4.9X per RIL, with an average of 0.72X, however, RIL219 has exceptionally low (0.002X) data (Supplementary Table 1). The SNPs identified (84,963) from resequencing of parental genotypes were compared with those identified from skim sequencing of RIL population; resulted in identifying 82,127 common SNPs. The remaining 2,836 SNPs could not be identified in RIL population. This might be because of low coverage sequencing of RIL population or because of errors in resequencing of parental genotypes. However, only 62,370 SNPs out of 82,127 which were located on pseudomolecules were considered for further analysis. These SNPs were further filtered, using the criteria of minimum allele frequency (MAF) of ≥0.20 and lines having ≥5% missing data being discarded, which resulted in 53,169 SNPs across 222 RILs being analysed. Additionally 54 SNPs within the “*QTL-hotspot*”^10^ were also included and a total of 53,223 SNPs were used for further study. The distribution of SNPs on the eight chickpea pseudomolecules is represented in Fig. 1 and Supplementary Table 2. The highest number of SNPs were identified on Ca4 (18,989 SNPs), while only 954 SNPs were identified on Ca5.

#### Recombination breakpoints and resolution of genes

**Figure 2:**
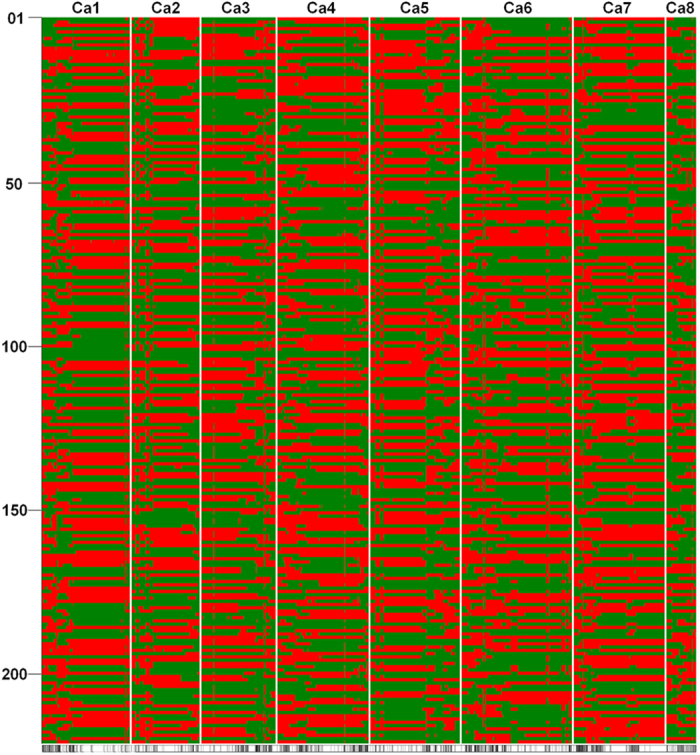
The recombination breakpoints identified in 222 recombinant inbred lines (RILs). A parent dependent 15 bp sliding window approach was used to identify true recombination breakpoints. A total of 53,223 SNPs identified were scored as “A” and “B” representing alleles from the two parents ICC 4958 and ICC 1882 respectively, and for each individual, the ratio of A and B alleles within the window was calculated using a perl script^18^. Windows with nine or more alleles from either parent were considered as homozygous for the respective region. The recombination break point was defined at the transition from one genotype to another. The chromosomes are labelled as Ca1 to Ca8 and are separated by vertical lines while each horizontal line represents a single RIL. Green and red bars represent segments from ICC 4958 and ICC 1882 genotypes, respectively. The number of bins per pseudomolecule ranged from 2.75 to 6.12 while an average of 35.71 bins were identified in an individual RIL. The black and white panel at the bottom indicates the consensus 1,610 bins identified in the entire RIL population.

**Figure 3:**
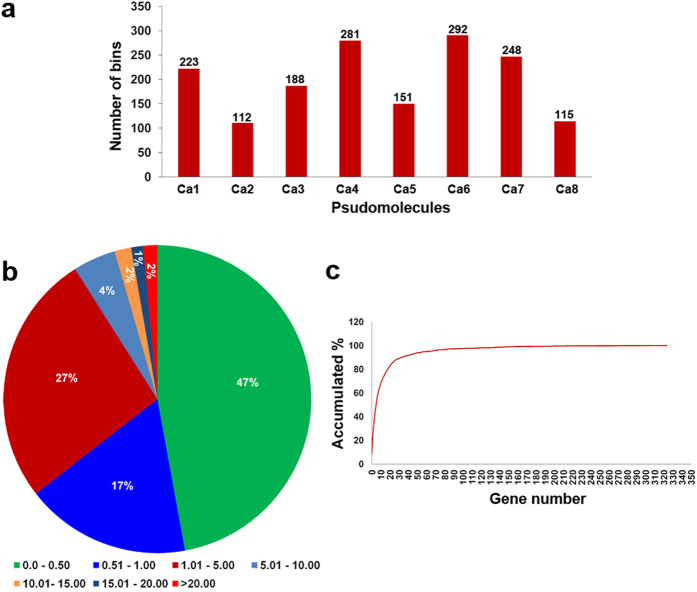
Features of recombination bin mapping in chickpea. (**a**) Distribution of the recombination bins on eight chickpea pseudomolecules. The number of recombination bins identified in each psudomolecule was depicted on top of each column. A minimum of 112 bins were identified on Ca2 while maximum 292 bins were identified on Ca6.; (**b**) Distribution of bin sizes identified in ICC 4958 × ICC 1882 population. Approximately, 50% of the bins were of ≤0.50 Mb size indicating majority of the recombination has been captured; (**c**) A plot of gene resolution. Number of genes in each recombination bin represented on the X-axis and the accumulated percentage of bins are on the Y-axis. In total, 8.68% (139) bins contained no gene, 14.29% (229) had only one gene, while 45.82% (734) had two to ten genes. Thus, 68.79% of bins had ≤10 genes.

By using the sliding window approach on 53,223 SNPs segregating in 222 RILs, a total of 1,610 bins were identified (Fig. 2 and Supplementary Table 2). The average number of bins identified in an individual RIL were 35.71, while the number of bins per pseudomolecule ranged from 2.75 to 6.12. The minimum number of bins (112) were identified on Ca2, whereas the highest number of bins (292) were identified on Ca6 (Figs 1 and 3a). The bin sizes ranged from 41 bp to 8.05 Mb, with an average of 210.60 Kb and median of 54.77 Kb. In total, 91% bins were of size ≤5 Mb while ~9% bins (144) had a size >5 Mb (Fig. 3b). By considering 28,269 genes in the chickpea genome^23^, the defined bin map covered 23,277 genes. In total, 8.68% (139) bins contained no gene, 14.29% (229) had only one gene, while 45.82% (734) had two to ten genes. Thus, 68.79% of bins had ≤10 genes (Fig. 3c).

#### Linkage map with bins as markers

All 1,610 bins, as mentioned above, were used as molecular markers for developing a genetic map (http://cegresources.icrisat.org/cmap/Chickpea_bin_map). Out of which, 1,557 (96.71%) bins could be integrated into a genetic map that spans 973.94 cM (Supplementary Table 2) and corresponds to 757.75 Kb/cM by considering the chickpea genome size of 738 Mb (http://cegresources.icrisat.org/cmap/Chickpea_bin_map). The average inter-bin interval per linkage group ranged from 0.45 cM (CaLG04) to 0.93 cM (CaLG02) with an overall average value of 0.66. The size of the linkage groups varied from 70.57 cM (CaLG08) to 196.27 cM (CaLG06) (Supplementary Table 2). The highest number of bins (286) were mapped on CaLG06, followed by CaLG04 (277), while the minimum number of bins were mapped on CaLG02 (103) (Supplementary Table 2). The order of bins on the genetic map were compared with their physical position on the chickpea genome sequence (CaGAv1.0). While excellent concordance was observed for linkage groups 4 and 8; minor differences were observed on other linkage groups (Supplementary Fig. 1).

### Identification of candidate genes in “*QTL-hotspot*” region

Combinations of two approaches, namely linkage-mapping based QTL analysis as well as genome-wide association study (GWAS) based gene enrichment analysis were undertaken.

### Bin mapping based QTL analysis

#### High-resolution mapping of QTLs

Genotyping data for 1,557 bins were analysed together with phenotyping data collected for 17 traits and two drought indices (drought tolerance index (DTI) and drought susceptibility index (DSI)) at 1–5 locations in 1–5 seasons, mentioned in our earlier study^5^. These data include: 4 root traits: root length density (RLD, cm cm^−3^); root dry weight (RDW, g); root surface area (RSA, cm^2^); root dry weight/total plant dry weight ratio (RTR, %), 6 yield and yield-related traits: pods/plant (POD); seeds/pod (SPD); 100-seed weight (100SDW, g); biomass (BM, g); harvest index (HI, %); yield (YLD, g), 4 morphological traits: shoot dry weight (SDW, g); plant height (PHT, cm); primary branches (PBS); secondary branches (SBS), 2 phenological traits: days to 50% flowering (DF); days to maturity (DM) and 1 physiological trait: delta carbon ratio (δ^13^C/DC). QTLs with >10% phenotypic variation explained (PVE) were considered as major QTLs while others were considered as minor QTLs. In summary, a total of 134 QTLs for 17 traits and two drought indices were identified, out of which 71 were major QTLs and 63 were minor QTLs (Supplementary Table 3, Supplementary Table 4). PVE for major QTLs ranged from 10.14 to 59.83% at 3.12 to 43.56 LOD value while for minor QTLs, PVE ranged from 4.75 to 9.85% at 3.00 to 4.91 LOD value.

**Figure 4:**
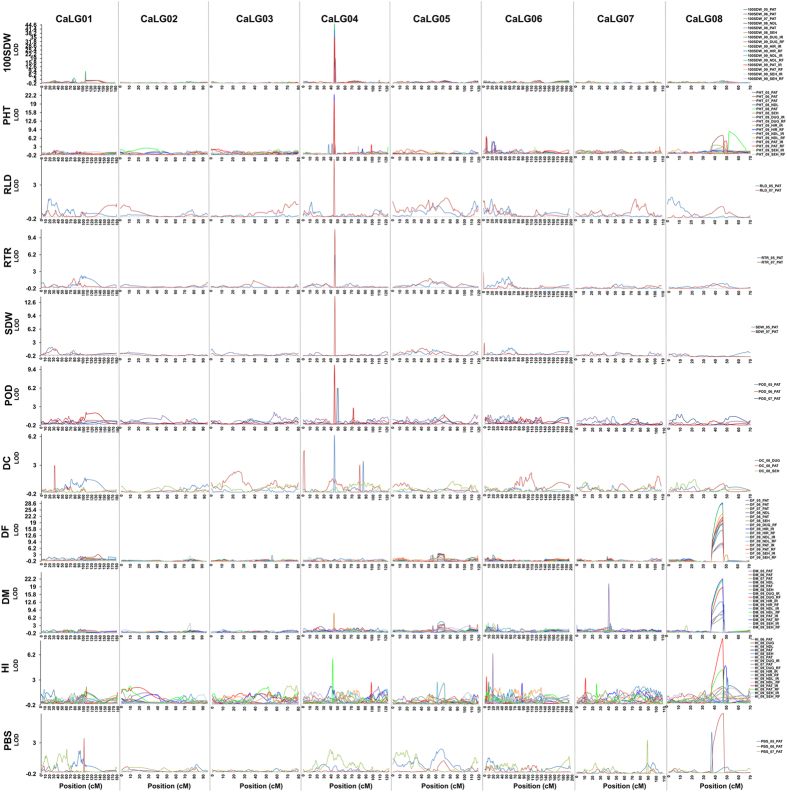
Genome-wide distribution of major QTLs identified for 11 traits. A total of 71 major QTLs identified for 11 traits (root length density (RLD, cm cm^−3^), root dry weight/total plant dry weight ratio (RTR, %), shoot dry weight (SDW, g), plant height (PHT, cm), primary branches (PBS), days to 50% flowering (DF), days to maturity (DM), pods/plant (POD), 100-seed weight (100SDW, g), harvest index (HI, %) and delta carbon ratio (DC)) have been shown. The linkage groups are separated by vertical lines, genetic distance is represented on the X-axis and LOD values are represented on Y-axis. Different coloured lines for each trait represent the phenotypic data collected over 1–5 seasons, 1–5 locations (Patancheru-PAT, Hiriyur-HIR, Nandyal-NDL, Durgapura-DUG and Sehore-SEH) and two environments (Rainfed-RF and Irrigated-IR). Major QTLs for 100SDW, PHT, RLD, RTR, SDW, POD and DC were identified on CaLG04; however, for traits like DF, DM, HI, and PBS major QTLs were identified on CaLG08.

Major QTLs were identified for 11 traits (RLD, RTR, SDW, PHT, PBS, DF, DM, POD, 100SDW, HI and DC) and minor QTLs were identified for all the analysed traits (Supplementary Table 3, Supplementary Table 4). The distribution of all the major QTLs in the chickpea genome has been shown in Fig. 4 and summarized in Supplementary Table 4. A total of 29 major QTLs for 9 traits (RLD, RTR, SDW, PHT, DM, POD, 100SDW, HI and DC) were observed on CaLG04 and that too within the “*QTL-hotspot*” region. On the other hand, 30 major QTLs for five traits (PHT, PBS, DF, DM and HI) were observed on CaLG08.

#### Splitting of “*QTL-hotspot*” region

**Figure 5:**
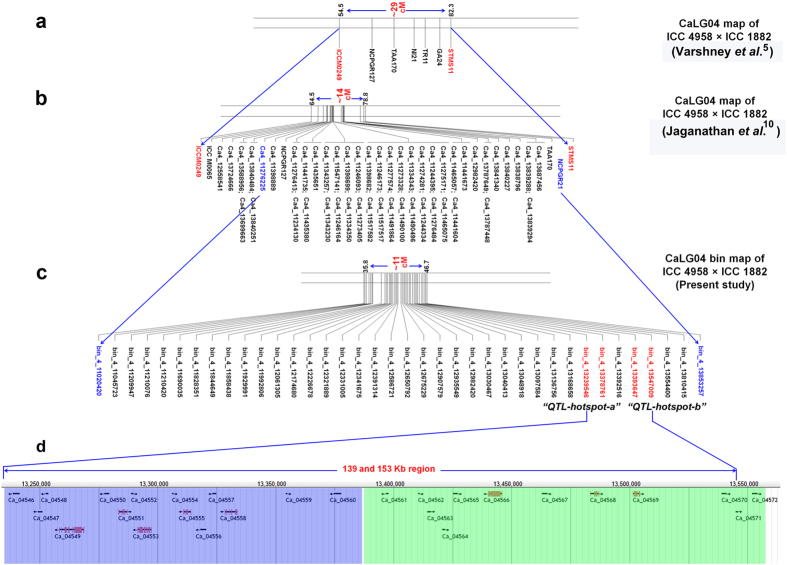
Refinement of “*QTL-hotspot*” region into “*QTL-hotspot_a*” and “*QTL-hotspot_b*” and identification of candidate genes. **(a**) The “*QTL-hotspot*” region, reported by Varshney and colleagues^5^, spanning 29 cM (corresponds 7.74 Mb on physical map) harbouring QTLs for several drought tolerance related traits on CaLG04; (**b**) Refined “*QTL-hotspot*” region (~14 cM corresponding to ca. 3 Mb on physical map) reported by Jaganathan *et al.*^10^ consisted of 49 SNPs and six SSRs; (**c**) Refined “*QTL-hotspot*” region on CaLG04 with newly integrated markers (recombination bins) from the current study. The markers, viz. bin_4_13853257 and bin_4_11020420 correspond to the refined 3Mb “*QTL-hotspot*” region reported earlier^10^. Integration of 1,421 SNPs to the “*QTL-hotspot*” region resulted in the identification of 38 recombination breakpoints and thereby split the “*QTL-hotspot*” region into “*QTL-hotspot_a*” (139.22 Kb; 0.23 cM) and “*QTL-hotspot_b*” (153.36 Kb; 0.22 cM). The “*QTL-hotspot_a*” was flanked by bin_4_13239546 and bin_4_13378761 while “*QTL-hotspot_b*” was flanked by bin_4_13393647 and bin_4_13547009. These four flanking markers were shown in the red colour font; (**d**) A ~300 Kb (13,239,546-13,547,009) snapshot of chickpea genome from JBrowse showing twenty six candidate genes identified in the “*QTL-hotspot_a*” and “*QTL-hotspot_b*” regions. A total of 15 genes (highlighted in blue colour area) were identified from “*QTL-hotspot_a*” while 11 candidate genes (highlighted in green colour area) were identified from “*QTL-hotspot_b*” region.

Although 1–17 major QTLs per trait were identified (Supplementary Table 4), we targeted the topmost QTL (a QTL which explained the highest phenotypic variation) for a given trait. As a result, 11 topmost QTLs with 10.14–59.83% PVE were selected for 11 traits (Table 1). While analysing flanking markers for these topmost QTLs, we identified two genomic regions, namely bin_4_13239546- bin_4_13378761 of 139.22 Kb and bin_4_13393647- bin_4_13547009 of 153.36 Kb sizes on CaLG04 containing the topmost QTLs for RLD, PHT, POD, 100SDW and DC; and for RTR and SDW, respectively (Table 1). Similarly, one genomic region (bin_8_6034209- bin_8_5984553) of 49.66 Kb size was identified on CaLG08 containing topmost QTLs for PBS, DF and HI (Table 1). However the topmost QTL for DM was present solitary on CaLG07. As expected, the two genomic regions on CaLG04 mentioned above were present within the “*QTL-hotspot*” region. In brief, this study split the “*QTL-hotspot*” region of ~3 Mb size into two smaller regions and we refer them as “*QTL-hotspot_a*” (bin_4_13239546- bin_4_13378761, equivalent to 139.22 Kb) and “*QTL-hotspot_b*” (bin_4_13393647- bin_4_13547009, equivalent to 153.36 Kb) (Fig. 5).

#### Candidate genes in “*QTL-hotspot*” sub-regions

**Figure 6:**
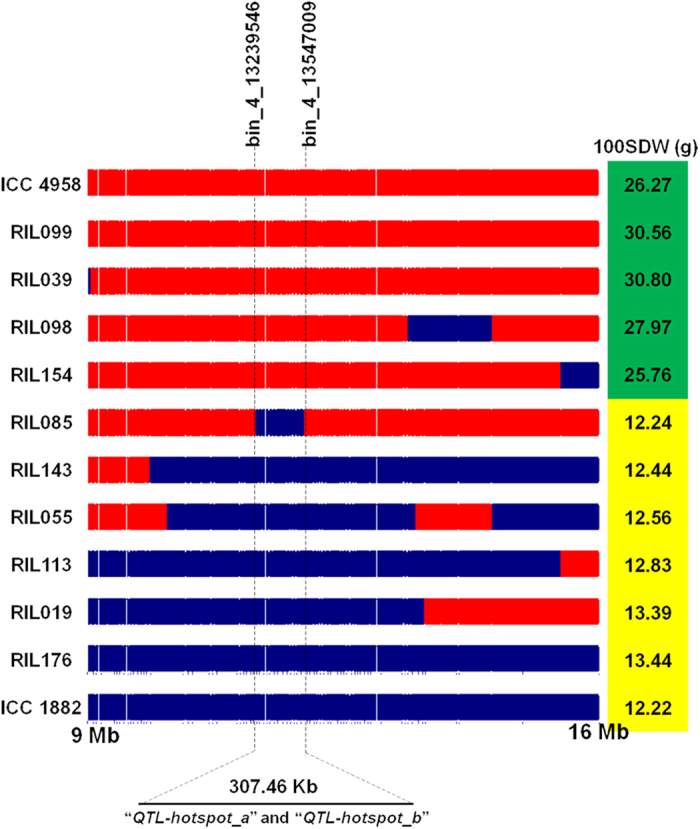
Fine mapping of “*QTL-hotspot*” for 100 seed weight (100SDW). The recombination breakpoints in “*QTL-hotspot*” region spanning 7 Mb size (9–16 Mb on physical map) among 4 recombinant inbred lines (RIL099, RIL038, RIL098, and RIL154) with high 100SDW, 6 RILs (RIL085, RIL143, RIL055, RIL113, RIL019, and RIL176) with low 100SDW and parental genotypes (ICC 4958 shown in red colour bars-high 100SDW, ICC 1882 shown in blue colour bars-low 100SDW) are shown. No recombination was observed within ~300 Kb refined region (“*QTL-hotspot_a*” and “*QTL-hotspot_b*”) in the case of RIL085 and other low and high RILs. This clearly indicates that the refined “*QTL-hotspot_a*” and “*QTL-hotspot_b*” are the candidate regions for 100SDW in chickpea.

It is evident from the results presented above that QTL analysis using high density bin mapping refined the “*QTL-hotspot*” region from 3 Mb^10^ to two sub-regions of 139.22 Kb (“*QTL-hotspot_a*”) and 153.36 Kb (“*QTL-hotspot_b*”). Comparison of these sub-regions with the chickpea genome assembly^23^ and genome annotations identified a total of 26 genes in these two regions. The “*QTL-hotspot_a*” sub-region has 15 and the “*QTL-hotspot_b*” contains 11 genes. Further, a detailed analysis of recombination at “*QTL-hotspot_a*” and “*QTL-hotspot_b*” along with the phenotypic data for 100SDW for 10 RILs (4 high and 6 low) clearly showed that the possible role of “*QTL-hotspot_a*” and “*QTL-hotspot_b*” to govern 100SDW trait in chickpea (Fig. 6).

### A priori gene enrichment analysis in the “*QTL-hotspot*” region

#### Genome-wide association study for Ca4 pseudomolecule

In order to identify candidate genes from the “*QTL-hotspot*” region present on pseudomolecule Ca4, 18,935 SNPs present on Ca4 and segregating in the RIL population were analysed with phenotyping data for all 17 traits and two drought indices for genome-wide association study. This analysis provided 3,574 marker-trait associations (MTAs) for all the 17 traits and two indices after applying bonferroni correction (Supplementary Table 5).

#### Candidate genes in “*QTL-hotspot*” region

While comparing the “*QTL-hotspot*” region with genomic positions of QTLs identified using bin map in the present study, a total of 34 QTLs including 29 major QTLs for 9 traits (RLD, RTR, SDW, PHT, DM, POD, 100SDW, HI and DC) and five minor QTLs for three traits (PHT, SBS and BM) were found in the region. Analysis of genomic positions for 34 QTLs showed 7 clusters in the “*QTL-hotspot*” region (Supplementary Table 3). Comparison of these 7 cluster regions with the chickpea genome and genome annotation data identified a total of 76 curated genes (Supplementary Table 6).

#### Promising candidate genes

To systematically summarize observed MTAs identified by GWAS and to identify candidate genes involved in the regulation of drought tolerance variation, probabilistic inference was made using the *PICARA* analytical pipeline. With the defined RMIP cutoff, significant associations were found for 6 traits viz. 100SDW, BM, DTI, PHT, POD and SDW (Supplementary Table 5) and 23 out of 76 genes showed significant enrichment for these traits. Detailed gene enrichment analysis has been summarized in Supplementary Table 6 while a representative image for 100SDW is shown in Supplementary Fig. 2.

### Prioritization and functional validation of candidate genes

As evident from above, bin mapping based QTL analysis identified 26 candidate genes in two sub-regions, while *a priori* gene enrichment analysis shortlisted 23 genes. Comparison of these gene sets prioritized 12 genes that were common in both analyses. Of these genes, 1 was present in “*QTL-hotspot_a*” and 11 were present in “*QTL-hotspot_b*” sub-regions. The functions of these genes were predicted by comparison with the NCBI non-redundant database using BLASTP (Supplementary Table 7). Various proteins such as serine threonine-protein kinase, E3 ubiquitin-protein ligase, homocysteine s-methyltransferase, leucine-rich repeat extensin-like along with uncharacterised mitochondrial and ribosomal proteins were identified.

**Figure 7:**
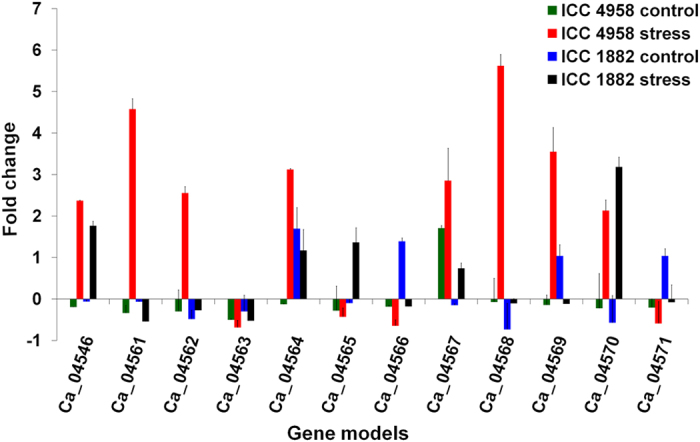
Gene expression analysis of 12 candidate genes identified using high density QTL mapping and gene enrichment analysis in control and stressed root tissues of ICC 4958 and ICC 1882 genotypes. QTL and gene enrichment analyses have identified 12 common candidate genes (1 from “*QTL-hotspot_a*” and 11 from “*QTL-hotspot_b*”). To study the expression of these genes under drought stress, root tissues of ICC 4958 (drought tolerant) and ICC 1882 (drought sensitive) genotypes were collected when the transpiration ratio reached 0.10 along with the respective controls and used for qRT-PCR analysis along with endogenous gene. The expression of each gene relative to endogenous gene was determined using 2^−∆∆Ct^ method while standard error was calculated based on expression values of three biological replicates. Among the 12 genes, 7 genes (Ca_04546, Ca_04561, Ca_04562, Ca_04564, Ca_04567, Ca_04568 and Ca_04569), were found to have a significantly higher expression in ICC 4958 than ICC 1882 under stress conditions. More specifically, Ca_04561, Ca_04562, Ca_04568 and Ca_04569 genes were found to be upregulated under stress condition in ICC 4958 while found to be down-regulated in ICC 1882 genotype as compared to respective control conditions.

**Figure 8:**
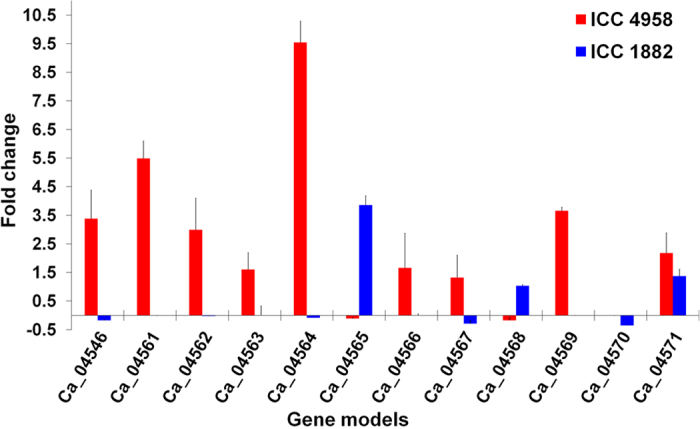
Differential gene expression profiles of 12 candidate genes in mature seeds of ICC 4958 and ICC 1882 genotypes. Expression of 12 genes in mature seeds of ICC 4958 (high 100SDW) and ICC 1882 (low 100SDW) was carried out to determine their role in controlling seed weight trait. All genes except Ca_04565, Ca_04568 and Ca_04570 were found to be significantly upregulated in ICC 4958 genotype compared to ICC 1882. Most of the upregulated genes showed no or negligible expression in ICC 1882 genotype. Genes such as Ca_04564, which encodes a ‘Leucine-rich repeat extensin-like protein’ showed about nine fold higher expression in ICC 4958 than ICC 1882.

For investigating functional implications of the prioritized 12 genes, differential gene expression profiling was undertaken in root tissues collected under stress and control conditions and in mature seeds of ICC 4958 and ICC 1882 using qRT-PCR. In root tissues, 7 genes, namely Ca_04546, Ca_04561, Ca_04562, Ca_04564, Ca_04567, Ca_04568 and Ca_04569 showed significantly higher expression in the ICC 4958 than ICC 1882 under stress conditions (Fig. 7). The expression level of these genes in ICC 4958 genotype was 0.61 to 5.72 fold higher under stress compared to that of ICC 1882. The Ca_04568 gene showed the highest (5.72 fold) change in expression followed by Ca_04561 (5.12 fold). In mature seeds, all genes except Ca_04565, Ca_04568 and Ca_04570 were found to be upregulated in the ICC 4958 genotype than ICC 1882 (Fig. 8). Interestingly, most of these upregulated genes were found not to be expressed or showed negligible expression in the ICC 1882 genotype. A maximum 9.63 fold higher expression was observed for the Ca_04564 gene, while the Ca_04571 gene showed only 0.80 fold higher expression in ICC 4958 as compared to ICC 1882.

Comparative analysis of expression of each gene in respective root and seed samples of ICC 4958 and ICC 1882 genotypes was also carried out. Four genes, viz. Ca_04561, Ca_04562, Ca_04567 and Ca_04569, were found to be upregulated in both root (under stress) and seed samples of ICC 4958 with a similar fold change in expression as compared to ICC 1882. For instance, the Ca_04561 gene was found 5.12 and 5.49 fold upregulated in stressed root tissue and mature seeds of ICC 4958 than the respective samples of ICC 1882. In contrast, gene Ca_04564 was found highly upregulated in seed (9.54 fold) as compared to the stressed root (3.12 fold) sample of ICC 4958 while significantly higher expression of Ca_04568 gene was observed in root (5.62) as compared to seed (−0.18) samples in ICC 4958 genotype.

## Discussion

NGS technologies have revolutionized the identification of candidate genes for crop improvement. Terminal drought is a major constraint to chickpea production and productivity, especially in arid and semi-arid regions. The changes in global climate are further predicted to aggravate losses due to drought in particular. In this context, the identification of candidate genes will be helpful to define breeding strategies and provide molecular markers to improve selection efficiency and therefore mitigate yield losses in chickpea. In this study, a skim sequencing approach was used and more than 53,000 SNPs were identified in a RIL population of ICC 4958 and ICC 1882 cross, which represents the largest number of markers used for QTL mapping in chickpea to date.

A sliding window based bin mapping approach identified an average of ~35 bins per RIL. Theoretically, each RIL contains 3–5 recombination events on each chromosome^24^. The results obtained match these assumptions (4 × 8 = 32). The average bin size was 210.60 Kb and more than 90% bins were less than 1Mb, suggesting that the majority of the recombinations were captured in this study. Moreover, ~68% bins contained ≤10 genes, indicating a high resolution of the bin map. All 1,610 recombination bins were used as markers to construct a linkage map of 973.94 cM. The average inter-bin distance (0.66) observed in this study was lower than other studies conducted on intra-specific mapping populations (0.94–7 cM) in chickpea^25^ indicating the current map is highly saturated. Such a highly saturated map can be used for targeted QTL mapping, QTL cloning and for identification of candidate genes for important agronomic traits in chickpea. We observed a high correlation between bin orders and their positions on the genome sequence for linkage groups 4 and 8. The interchanging of bin positions observed on other linkage groups could be due to misassembled portions of chickpea genome assembly. Such misassembled regions in chickpea genome have recently been reported^26^,^27^.

The majority of QTLs identified in the present study are in concordance with earlier studies^5^,^10^. For instance, in all the three studies, QTLs for 100SDW, RLD, PHT, POD, RTR and SDW were observed on CaLG04, while a QTL for DF was reported to be present on CaLG08 indicating the quality and accuracy of the linkage map. The “*QTL-hotspot*” region of 7.74 Mb identified in earlier studies^5^ had 7 SSRs and was further refined to ca. 3 Mb by integration of 49 SNP markers^10^. The present study integrated 1,421 SNPs and identified 38 recombination breakpoints within this region, thereby splits the “*QTL-hotspot*” into two subregions viz. “*QTL-hotspot_a*” (139.22 Kb; 0.23 cM) and “*QTL-hotspot_b*” (153.36 Kb; 0.22 cM), and thus further narrowed down the earlier reported “*QTL-hotspot*” region to a size appropriate for candidate gene identification. This demonstrates that WGRS approach helps to significantly improve the accuracy and resolution of QTL mapping similar to what has been observed in several studies in other crops. For example, in soybean, QTLs of sizes 7.90 to 340 Kb were identified for root knot nematode resistance using ~100 thousand SNPs and 242 RILs^20^, while in another study^21^ QTLs of 176 Kb to 1.28 Mb sizes were identified using 1,798,504 SNPs and 96 ILs for salt tolerance. This resolution is equivalent to traditional fine mapping studies. For instance, a grain-weight QTL, *gw*3.1 in rice was narrowed down to 93.8 Kb using fine mapping procedures^28^. These studies suggest that the bin mapping approach can be used for candidate gene identification using a RIL population and thus avoiding the laborious time required for traditional fine mapping.

A sequential approach of QTL and gene enrichment analysis identified 12 candidate genes for drought tolerance related traits in chickpea. Annotation of these genes identified several genes, including E3 ubiquitin-protein ligase, leucine-rich repeat extensin-like protein, serine threonine-protein kinase, homocysteine s-methyltransferase, vicilin, which have been reported to play a role in biotic and abiotic stress tolerance^29^,^30^,^31^,^32^ and differential gene expression analysis proved their involvement in the drought tolerance mechanism in chickpea. Ca_04568 gene was annotated as ‘Homocysteine s-methyltransferase’ and found to be upregulated in ICC 4958 genotypes only in root tissue under stress conditions indicating tissue specific expression. ‘Homocysteine s-methyltransferase’ gene was also reported to be upregulated in drought tolerant chickpea cultivar ‘Xj-209’ under stress condition^32^ and could be one of the important genes involved in drought tolerance mechanism in chickpea. Proteomic analysis of rice seedlings also reported involvement of ‘Homocysteine s-methyltransferase’ in regulating cold stress^33^. The Ca_04567 gene was annotated as ‘LRR receptor like serine threonine-protein kinases’ which are the well characterized membrane proteins known for their roles in stress response and abscisic acid (ABA) regulation^29^. More than 2 fold higher expression of this gene was observed in the drought tolerant genotype, ICC 4958 than the drought sensitive genotype ICC 1882, suggesting a role in drought tolerance in chickpea. Additionally, the Ca_04561 gene, which is predicted to be an ‘E3 ubiquitin-protein ligase’, was up-regulated in ICC 4958 while down-regulated in ICC 1882 under stress conditions. Ubiquitination is an important post translational modification to regulate growth and development in all eukaryotes. Various studies in plants^34^,^35^,^36^ reported the role of the ubiquitin 26S proteasome system (UPS) in regulating fundamental processes such as embryogenesis, photomorphogenesis, and organ development. E3 ubiquitin ligase, which determines substrate specificity, is an important enzyme of the ubiquitination pathway^37^,^38^. Recent studies^39^,^40^,^41^ reported the involvement of E3 ubiquitin ligase in the regulation of abiotic stress tolerance. A study in *Arabidopsis*^42^ showed that suppression of one of RING E3 ubiquitin ligase (*AtATL78*) gene was responsible for increasing tolerance to cold stress while decreasing drought stress tolerance. The regulation of this gene could be responsible for determining drought sensitivity/resistance in chickpea. Interestingly, this gene showed higher expression in mature seeds of ICC 4958 (larger seed size) as compared to ICC 1882 (smaller seed size), although, a loss of function of RING-type E3 ubiquitin ligase was reported to be responsible for enhanced grain width, weight and yield in rice^31^. This indicates that ‘E3 ubiquitin-protein ligase’ could be responsible for only abiotic stress tolerance in chickpea while a different mechanism might be present for controlling seed size. Ca_04564 gene was identified as ‘leucine-rich repeat extensin-like protein’. This gene was found highly over-expressed in ICC 4958 seed while showing negligible expression in ICC 1882 seeds. The role of extensin like genes in seed germination has already been reported in *Arabidopsis*^43^. They found extensin like genes are expressed specifically in endosperm and might play a role in cell wall modification. The present study suggests a detailed study of such genes in the context of seed size/weight. Along with the above genes, one uncharacterised mitochondrial protein showing higher expression in both stressed root and seed samples of ICC 4958, was also identified. This suggests a combined action of different genes may be responsible for drought tolerance in ICC 4958. However, we did not observe any sequence level variation for these genes between tolerant and sensitive genotypes suggesting detailed characterization of these genes could help to provide deeper insights on their role in drought tolerance mechanism in chickpea.

The present study showed that bin mapping based QTL analysis approach is advantageous over traditional QTL study. However, the resolution required to identify gene(s) for complex traits could not be achieved using the bi-parental mapping population because of inherent limitation of low recombination with limited population size. In such cases, developing a large scale population will help to capture more recombinations, thereby, increasing the resolution to dissect the complex traits to the gene level.

In summary, a bin mapping approach was successfully applied to identify 1,610 recombination breakpoints in chickpea. A linkage map of 973.94 cM was constructed using recombination bins as markers and this was used for QTL analysis of drought related traits. The QTL study identified 71 major QTLs and delineates the “*QTL-hotspot*” region from ca. 3 Mb to two QTL regions viz. “*QTL-hotspot_a*” of 139.22 Kb and “*QTL-hotspot_b*” of 153.36 Kb sizes. Further, bin mapping and gene enrichment analysis identified a set of 12 candidate genes. Some of the identified genes such as E3 ubiquitin ligase, Serine threonine protein kinases, homocysteine s-methyltransferase were annotated as candidate drought tolerance genes, and expression profiling suggests their involvement in drought tolerance in chickpea. Detailed analysis of these genes will help to further elucidate drought tolerance mechanism in chickpea.

## Methods

### Plant material

A set of 232 RILs was developed using the single seed decent method by crossing ICC 4958 (a drought tolerant genotype) and ICC 1882 (a drought sensitive genotype). Phenotypic data for 17 different traits along with two drought indices (DTI and DSI) described in our earlier study^5^ were used for identification of QTLs.

Genomic DNA was isolated from parental genotypes and RILs using high throughput mini-DNA extraction method as described earlier^44^. The quality and quantity of DNA was checked using a spectrophotometer (Shimadzu UV160A, Japan).

### Library construction, skim sequencing and SNP calling

For the RILs and parental lines of the mapping population, a total of 50 ng DNA from each sample was used to prepare an Illumina Nextera library according to the manufacturer’s instructions. In brief, the genomic DNA was simultaneously fragmented and tagged with sequencing adaptors using an engineered transposome. The fragments were then amplified using limited PCR cycles which also adds index sequences on both ends of the DNA, thus enabling dual-indexed sequencing of pooled libraries. Libraries for 96 individuals were pooled and sequenced on the Illumina HiSeq 2500 platform. Parental genotypes were sequenced separately at high sequencing depth (~8X) whereas RILs were sequenced at low coverage of ~0.72X (average of RILs).

The reads obtained were filtered to remove low quality bases and used for SNP identification. Initially, the reads from the parental genotypes (ICC 4958 and ICC 1882) were aligned to the draft chickpea genome (CaGAv1.0) using SOAP^45^. Uniquely mapped reads were considered for SNP calling using SGSAutoSNP software^46^ with default parameters. Similarly, the low quality reads obtained from RILs were filtered out and SNPs were identified from the remaining reads. The identified SNPs were filtered with a minor allele frequency (MAF) cutoff of 0.2 and lines with ≥5% missing data were also excluded. SNPs were scored as “A” and “B” representing alleles from the two parents ICC 4958 and ICC 1882 respectively.

### Identification of recombination break points

A 15 bp sliding window approach was used to predict recombination break points^18^. This was determined based on the ratio of the alleles in the sliding window using a perl script^18^. The script was used with necessary changes according to chickpea genome co-ordinates. For each individual, the ratio of A and B alleles within the window was calculated. Windows with nine or more alleles from either parent were considered as homozygous for an individual. The recombination break point was defined at the transition from one genotype to another. The recombination breakpoints obtained from all the individual RILs were aligned and compared over 100 Kb intervals. The successive intervals lacking recombination break points within an entire population were combined and considered as a single bin. These bins were then used as markers for linkage map construction.

### Construction of bin map and QTL analysis

The bins were used as genetic markers for the construction of a linkage map. Map construction was carried out using QTL IciMapping Version 3.3 software^47^. The REcombination Counting and ORDering (RECORD) algorithm was used for marker ordering. Sum of adjacent criterion (SAD) ripple was performed to confirm the marker order. The marker order and their positions on genetic and physical map were visualized using Strudel V. 1.12.03.20^48^.

QTL IciMapping software was also used with the phenotype data for 17 drought related traits and two drought indices and bin map for QTL analysis. The ICIM-ADD mapping method, along with other default parameters, was used. The LOD threshold was set by using permutation 1000 and p value ≤ 0.05. The results were compared with earlier studies^5^,^10^ to determine the quality and accuracy of the bin map. Further, the QTL results were used to refine the earlier identified “*QTL-hotspot*” region and also to identify candidate genes controlling respective traits.

### Gene enrichment analysis

*PICARA* enrichment algorithm delineates local linkage structure and provides a probabilistic inference of GWAS signal enrichment that defines the significance of co-localization of marker-trait association and curated candidate genes^49^. To generate the local linkage blocks, all SNPs on CaLG04 were included in the pairwise linkage disequilibrium analysis by Lewontin’s scaled D’ method^50^. Only significant pairwise LD estimates were preserved for calculating a local linkage block. For the candidate genes located in SNP poor regions, where no SNPs within and flanking candidate gene can be located, a chromosome-wide estimation was calculated by taking the median physical distance in base-pairs between all SNP pairs that are in complete dependency (D’ = 1).

For each *a priori* candidate gene, *PICARA* enrichment estimates the amount of co-localizing GWAS signal with that gene, compares the weighted probability of co-localization with a permutation test from a chromosome-wide resampling and a significance test score was generated following a hypergeometric distribution^49^. A prior probability of co-localization with significant association variants was generated by weighting the cumulative probability between the given candidate gene and all SNPs from the same chromosome with RMIP (re-sampling model inclusion probability^51^) cutoffs. In this study, a RMIP ≥ 5, which is the same SNP variant showing at least 5 times an association in 100 subsets of resampling, was used as the cutoff for association significance. The permutation was done through 10,000 randomized association sets each containing the same number of associations as the true data, sampled without replacement from all SNPs on the chromosome. Association significance (in this case, RMIP values) was assigned to each set of the 10,000 associations from the true data, and compared to their posterior probability of co-localization with the *a priori* candidate genes. The vector of 10,000 significance results, then served the null distribution of co-localization.

### Quantitative gene expression analysis

Quantitative real time PCR (qRT-PCR) analysis was carried out to study the expression of candidate genes in RNAs isolated from seed (for seed weight trait) and root (for drought tolerance) tissues harvested under control as well as drought challenged conditions. For drought, a slow drought experiment for both ICC 4958 and ICC 1882 was conducted under greenhouse conditions, as described in Ray *et al.*^52^. Root samples of the selected genotypes were collected when the transpiration ratio reached 0.10 along with the respective controls. Each treatment was maintained in 3 biological replications. For expression profiling, root tissues of drought tolerant ICC 4958 and sensitive ICC 1882 genotypes under control and drought conditions were collected. Tissues were washed thoroughly with 0.10% DEPC water, frozen in liquid nitrogen and stored at −80 °C. Total RNA was extracted from the harvested tissues using TRIzol (Invitrogen, USA) according to the manufacturer’s protocol. RNA quality was assessed on 1.20% formaldehyde agarose gels, while the purity of RNA was assessed using a Nanovue spectrophotometer (A260/A280 ratio). First strand cDNA was synthesized from total RNA (2.50 μg) using a cDNA synthesis kit (Superscript® III; Invitrogen, USA) following manufacturer’s instructions. For seed weight trait, the mature seeds of ICC 4958 and ICC 1882 were soaked in 0.10% DEPC treated water for 30 minutes before RNA extraction. The same protocol mentioned above was used for total RNA extraction and first strand cDNA synthesis.

Gene-specific primers for qRT-PCR were designed using primer 3 software^53^ and used for PCR amplification using the conditions described in^54^. qRT-PCR was performed using an Applied Biosystems 7500 Real Time PCR System with SYBR green chemistry (Applied Biosystems, USA) according to the manufacturer’s instructions. The data from different PCR runs or cDNA samples were compared by using the mean of the CT values of the three biological replicates that were normalized to the mean CT values of the endogenous gene, glyceraldehyde 3-phosphate dehydrogenase (GAPDH). The relative expression ratios were determined using the 2^_∆∆Ct^ method and student’s t-test was used to calculate significance^55^.

## Additional Information

**How to cite this article**: Kale, S. M. *et al.* Prioritization of candidate genes in “*QTL-hotspot*” region for drought tolerance in chickpea (*Cicer arietinum* L.). *Sci. Rep.* **5**, 15296; doi: 10.1038/srep15296 (2015).

## Supplementary Material

Supplementary Tables 1-7

Supplementary Figures 1-2

## Figures and Tables

**Table 1:** Comparative analysis of topmost QTLs identified for drought related traits in chickpea from the RIL population of ICC 4958 × ICC 1882 cross using recombination bins as markers (Present study), SSRs (Varshney, *et al.* 5) and SSRs and SNPs (Jaganathan, *et al.* 10).

Trait name	Present study							Varshney*et al.*^5^				Jaganathan *et al.*^10^			
	Linkage groups	Flanking markers	Regions	Physical size (Kb)	Genetic size (cM)	LOD	PVE (%)	Linkage groups	Flanking markers	LOD	PVE (%)	Linkage groups	Flanking markers	LOD	PVE (%)
RLD	CaLG04	bin_4_13239546 - bin_4_13378761	region1	139.22	0.23	5.23	10.36	CaLG04	NCPGR127 - NCPGR21	4.78	10.90	CaLG04	ICCM0065 - Ca4_11276225	4.59	12.09
PHT	CaLG04	bin_4_13239546 - bin_4_13378761	region1	139.22	0.23	24.34	41.76	CaLG04	NCPGR127 - NCPGR21	16.55	30.20	CaLG04	Ca4_12982420 - TAA170	29.16	34.57
POD	CaLG04	bin_4_13239546 - bin_4_13378761	region1	139.22	0.23	9.82	16.66	CaLG04	NCPGR127 - NCPGR21	13.14	23.18	CaLG04	Ca4_13687456 - TAA170	18.79	32.34
100SDW	CaLG04	bin_4_13239546 - bin_4_13378761	region1	139.22	0.23	43.56	59.83	CaLG04	NCPGR127 - NCPGR21	32.38	58.20	CaLG04	Ca4_13687456 - TAA170	50.13	60.41
DC	CaLG04	bin_4_13239546 - bin_4_13378761	region1	139.22	0.23	6.11	11.90	CaLG04	NCPGR127 - NCPGR21	3.54	9.38	—	—	—	—
RTR	CaLG04	bin_4_13393647 - bin_4_13547009	region 2	153.36	0.22	10.57	20.09	CaLG04	TAA170 - NCPGR21	7.59	16.67	CaLG04	Ca4_13840227 - NCPGR21	8.96	13.56
SDW	CaLG04	bin_4_13393647 - bin_4_13547009	region 2	153.36	0.22	13.78	25.22	CaLG04	TAA170 - NCPGR21	7.97	17.59	CaLG04	Ca4_13840227 - TAA170	16.86	26.91
PBS	CaLG08	bin_8_6034209 - bin_8_5984553	region 3	49.66	9.47	5.70	11.27	CaLG06	TA106 - H1I16	3.01	8.73	CaLG08	CaM0812 - NCPGR164	5.27	12.92
DF	CaLG08	bin_8_6034209 - bin_8_5984553	region 3	49.66	9.47	28.19	44.76	CaLG08	NCPGR164 - CaM1918	9.63	26.87	CaLG08	NCPGR164 - Ca8_3050452	46.03	67.71
HI	CaLG08	bin_8_6034209 - bin_8_5984553	region 3	49.66	9.47	8.01	15.42	CaLG01	cpPb-679915 - CaM0393	4.96	14.36	CaLG08	NCPGR164 - Ca8_3050452	14.52	25.94
DM	CaLG07	bin_7_12870961 - bin_7_12856579	—	14.38	0.23	19.53	45.38	CaLG04	NCPGR127 - TAA170	8.75	19.71	CaLG08	NCPGR164 - Ca8_3050452	30.01	47.43

In present study, two regions viz. “*QTL-hotspot_a*” (region1; bin_4_13239546 - bin_4_13378761) and “*QTL-hotspot_b*” (region2; bin_4_13393647 - bin_4_13547009) were identified on CaLG04 harbouring QTLs for seven traits.

## References

[b1] Jukanti A. K., Gaur P. M., Gowda C. L. L. & Chibbar R. N. Nutritional quality and health benefits of chickpea (*Cicer arietinum* L.): a review. Brit. J. Nutr. 108, S11–S26 (2012). 22916806 10.1017/S0007114512000797

[b2] Ahmad F., Gaur P. M. & Croser J. Chickpea (*Cicer arietinum* L.). In: Genetic Resources, Chromosome Engineering, and Crop Improvement—Grain Legumes (eds Singh R. J., Jauhar P. P.). CRC Press (2005).

[b3] Hamwieh A., Imtiaz M. & Malhotra R. S. Multi-environment QTL analyses for drought-related traits in a recombinant inbred population of chickpea (*Cicer arientinum* L.). Theor. Appl. Genet. 126, 1025–1038 (2013). 23283512 10.1007/s00122-012-2034-0

[b4] Rehman A. U. *et al.* Mapping QTL associated with traits affecting grain yield in chickpea (*Cicer arietinum* L.) under terminal drought stress. Crop Sci. 51, 450–463 (2011).

[b5] Varshney R. K. *et al.* Genetic dissection of drought tolerance in chickpea (*Cicer arietinum* L.). Theor. Appl. Genet. 127, 445–462 (2014). 24326458 10.1007/s00122-013-2230-6 PMC3910274

[b6] Roorkiwal M. *et al.* Allele diversity for abiotic stress responsive candidate genes in chickpea reference set using gene based SNP markers. Front. Plant Sci. 5, 10.3389/fpls.2014.00248 (2014). PMC4046317 24926299

[b7] Thudi M. *et al.* Genetic dissection of drought and heat tolerance in chickpea through genome-wide and candidate gene-based association mapping approaches. PLoS One 9, e96758, (2014). 24801366 10.1371/journal.pone.0096758 PMC4011848

[b8] Varshney R. K. *et al.* Fast-track introgression of “*QTL-hotspot*” for root traits and other drought tolerance traits in JG 11, an elite and leading variety of chickpea. *Plant* Genome 6, 10.3835/plantgenome2013.07.0022 (2013).

[b9] Varshney R. K. *et al.* Integrated physical, genetic and genome map of chickpea (*Cicer arietinum* L.). Funct. Integr. Genomic 14, 59–73 (2014). 10.1007/s10142-014-0363-6 PMC4273598 24610029

[b10] Jaganathan D. *et al.* Genotyping-by-sequencing based intra-specific genetic map refines a *“QTL-hotspot*” region for drought tolerance in chickpea. Mol. Genet. Genomics 290, 559–571 (2015). 25344290 10.1007/s00438-014-0932-3 PMC4361754

[b11] Craig D. W. *et al.* Identification of genetic variants using bar-coded multiplexed sequencing. Nat. Methods 5, 887–893 (2008). 18794863 10.1038/nmeth.1251 PMC3171277

[b12] Cronn R. *et al.* Multiplex sequencing of plant chloroplast genomes using Solexa sequencing-by-synthesis technology. Nucleic Acids Res. 36, e122 (2008). 18753151 10.1093/nar/gkn502 PMC2577356

[b13] Elshire R. J. *et al.* A robust, simple genotyping-by-sequencing (GBS) approach for high diversity species. PLoS One 6, e19379, (2011). 21573248 10.1371/journal.pone.0019379 PMC3087801

[b14] Deschamps S., Nannapaneni K., Zhang Y. & Hayes K. Local assemblies of paired end reduced representation libraries sequenced with the illumina genome analyser in maize. Int. J. Plant Genomics 2012, 8 10.1155/2012/360598 (2012). PMC3474217 23093955

[b15] Poland J. A. & Rife T. W. Genotyping-by-sequencing for plant breeding and genetics. Plant Genome 5, 92–102 (2012).

[b16] Chen Z. L. *et al.* An ultra-high density bin-map for rapid QTL mapping for tassel and ear architecture in a large F-2 maize population. BMC Genomics 15, 10.1186/1471-2164-15-433 (2014). PMC4059873 24898122

[b17] Golicz A. A., Bayer P. E. & Edwards D. Skim-based genotyping by sequencing. Methods Mol. Biol. 1245, 257–270 (2015). 25373763 10.1007/978-1-4939-1966-6_19

[b18] Huang X. H. *et al.* High-throughput genotyping by whole-genome resequencing. Genome Res. 19, 1068–1076 (2009). 19420380 10.1101/gr.089516.108 PMC2694477

[b19] Sasaki A. *et al.* Green revolution: A mutant gibberellin-synthesis gene in rice—New insight into the rice variant that helped to avert famine over thirty years ago. Nature 416, 701–702 (2002). 11961544 10.1038/416701a

[b20] Xu X. Y. *et al.* Pinpointing genes underlying the quantitative trait loci for root-knot nematode resistance in palaeopolyploid soybean by whole genome resequencing. P. Natl. Acad. Sci. USA 110, 13469–13474 (2013). 10.1073/pnas.1222368110 PMC3746920 23898176

[b21] Qi X. P. *et al.* Identification of a novel salt tolerance gene in wild soybean by whole-genome sequencing. Nat. Commun. 5, 1–11 (2014). 10.1038/ncomms5340 PMC4104456 25004933

[b22] Xie W. B. *et al.* Parent-independent genotyping for constructing an ultrahigh-density linkage map based on population sequencing. P. Natl. Acad. Sci. USA 107, 10578–10583 (2010). 10.1073/pnas.1005931107 PMC2890813 20498060

[b23] Varshney R. K. *et al.* Draft genome sequence of chickpea (*Cicer arietinum*) provides a resource for trait improvement. Nat. Biotechnol. 31, 240–246 (2013). 23354103 10.1038/nbt.2491

[b24] Lai J. S. *et al.* Genome-wide patterns of genetic variation among elite maize inbred lines. Nat. Genet. 42, 1027–U1158 (2010). 20972441 10.1038/ng.684

[b25] Kujur A. *et al.* Ultra-high density intra-specific genetic linkage maps accelerate identification of functionally relevant molecular tags governing important agronomic traits in chickpea. Sci. Rep. 5, 9468, 10.1038/srep09468 (2015). 25942004 PMC5386344

[b26] Bayer P. E. *et al.* High resolution skim genotyping by sequencing reveals the distribution of crossovers and gene conversions in *Cicer arietinum* and *Brassica napus*. Theor. Appl. Genet. 128, 1039–1047 (2015). 25754422 10.1007/s00122-015-2488-y

[b27] Ruperao P. *et al.* A chromosomal genomics approach to assess and validate the desi and kabuli draft chickpea genome assemblies. Plant Biotechnol. J. 12, 778–786 (2014). 24702794 10.1111/pbi.12182

[b28] Li J. M., Thomson M. & McCouch S. R. Fine mapping of a grain-weight quantitative trait locus in the pericentromeric region of rice chromosome 3. Genetics 168, 2187–2195 (2004). 15611185 10.1534/genetics.104.034165 PMC1448733

[b29] Osakabe Y., Yamaguchi-Shinozaki K., Shinozaki K. & Tran L. S. P. Sensing the environment: key roles of membrane-localized kinases in plant perception and response to abiotic stress. J. Exp. Bot. 64, 445–458 (2013). 23307915 10.1093/jxb/ers354

[b30] Ruiz J. M. & Blumwald E. Salinity-induced glutathione synthesis in *Brassica napus*. Planta 214, 965–969 (2002). 11941474 10.1007/s00425-002-0748-y

[b31] Song X. J., Huang W., Shi M., Zhu M. Z. & Lin H. X. A QTL for rice grain width and weight encodes a previously unknown RING-type E3 ubiquitin ligase. Nat. Genet. 39, 623–630 (2007). 17417637 10.1038/ng2014

[b32] Wang X. S. *et al.* Transcriptional responses to drought stress in root and leaf of chickpea seedling. Mol. Biol. Rep. 39, 8147–8158 (2012). 22562393 10.1007/s11033-012-1662-4

[b33] Hashimoto M. & Komatsu S. Proteomic analysis of rice seedlings during cold stress. Proteomics 7, 1293–1302 (2007). 17380535 10.1002/pmic.200600921

[b34] Pokhilko A. *et al.* Ubiquitin ligase switch in plant photomorphogenesis: A hypothesis. J. Theor. Biol. 270, 31–41 (2011). 21093457 10.1016/j.jtbi.2010.11.021 PMC3021735

[b35] Sonoda Y. *et al.* Regulation of leaf organ size by the *Arabidopsis* RPT2a 19S proteasome subunit. Plant J. 60, 68–78 (2009). 19500299 10.1111/j.1365-313X.2009.03932.x

[b36] Thomann A. *et al.* *Arabidopsis CUL3A* and *CUL3B* genes are essential for normal embryogenesis. Plant J. 43, 437–448 (2005). 16045478 10.1111/j.1365-313X.2005.02467.x

[b37] Lee J. H. & Kim W. T. Regulation of abiotic stress signal transduction by E3 ubiquitin ligases in *Arabidopsis*. Mol. Cells 31, 201–208 (2011). 21347703 10.1007/s10059-011-0031-9 PMC3932693

[b38] Lyzenga W. J. & Stone S. L. Abiotic stress tolerance mediated by protein ubiquitination. J. Exp. Bot. 63, 599–616 (2012). 22016431 10.1093/jxb/err310

[b39] Ju H. W., Min J. H., Chung M. S., Kim C. S. The *atrzf1* mutation of the novel RING-type E3 ubiquitin ligase increases proline contents and enhances drought tolerance in *Arabidopsis*. Plant Sci. 203, 1–7 (2013). 23415322 10.1016/j.plantsci.2012.12.007

[b40] Kim S. J. & Kim W. T. Suppression of *Arabidopsis* RING E3 ubiquitin ligase *AtATL78* increases tolerance to cold stress and decreases tolerance to drought stress. Febs Lett. 587, 2584–2590 (2013). 23831064 10.1016/j.febslet.2013.06.038

[b41] Li J. H. *et al.* The E3 ligase AtRDUF1 positively regulates salt stress responses in *Arabidopsis thaliana*. PLoS One 8, e71078, (2013). 23951086 10.1371/journal.pone.0071078 PMC3741333

[b42] Kim J. H. & Kim W. T. The *Arabidopsis* RING E3 ubiquitin ligase AtAIRP3/LOG2 participates in positive regulation of high-salt and drought stress responses. Plant Physiol. 162, 1733–1749 (2013). 23696092 10.1104/pp.113.220103 PMC3707541

[b43] Dubreucq B. *et al.* The *Arabidopsis AtEPR1* extensin-like gene is specifically expressed in endosperm during seed germination. Plant J. 23, 643–652 (2000). 10972890 10.1046/j.1365-313x.2000.00829.x

[b44] Cuc L. M. *et al.* Isolation and characterization of novel microsatellite markers and their application for diversity assessment in cultivated groundnut (*Arachis hypogaea*). BMC Plant Biol. 8, 10.1186/1471-2229-8-55 (2008). PMC2416452 18482440

[b45] Li R. *et al.* SOAP2: an improved ultrafast tool for short read alignment. Bioinformatics 25, 1966–1967 (2009). 19497933 10.1093/bioinformatics/btp336

[b46] Lorenc M. T. *et al.* Discovery of single nucleotide polymorphisms in complex genomes using SGSautoSNP. Biology 1, 370–382 (2012). 24832230 10.3390/biology1020370 PMC4009776

[b47] Meng L., Li H., Zhang L. & Wang J. QTL IciMapping: Integrated software for genetic linkage map construction and quantitative trait locus mapping in biparental populations. Crop J. 3, 269–283 (2015).

[b48] Bayer I. *et al.* Comparative visualization of genetic and physical maps with Strudel. Bioinformatics 27, 1307–1308 (2011). 21372085 10.1093/bioinformatics/btr111 PMC3077070

[b49] Chen C. *et al.* *PICARA*, an analytical pipeline providing probabilistic inference about *a priori* candidates genes underlying genome-wide association QTL in plants. PLoS One 7, e4659, (2012). 10.1371/journal.pone.0046596 PMC3492367 23144785

[b50] Lewontin R. C. The interaction of selection and linkage. I. General considerations; heterotic models. Genetics 49, 49–67 (1964). 17248194 10.1093/genetics/49.1.49 PMC1210557

[b51] Tian F. *et al.* Genome-wide association study of leaf architecture in the maize nested association mapping population. Nat. Genet. 43, 159–U113 (2011). 21217756 10.1038/ng.746

[b52] Ray J. D. & Sinclair T. R. The effect of pot size on growth and transpiration of maize and soybean during water deficit stress. J. Exp. Bot. 49, 1381–1386 (1998).

[b53] Rozen S. & Skaletsky H. Primer3 on the WWW for general users and for biologist programmers. Methods Mol. Biol. 132, 365–386 (2000). 10547847 10.1385/1-59259-192-2:365

[b54] Mir R. R. *et al.* Candidate gene analysis for determinacy in pigeonpea (*Cajanus* spp.). Theor. Appl. Genet. 127, 2663–2678 (2014). 25331300 10.1007/s00122-014-2406-8 PMC4236620

[b55] Livak K. J. & Schmittgen T. D. Analysis of relative gene expression data using realtime quantitative PCR and the 2^−∆∆Ct^ method. Methods 25, 402–408 (2001). 11846609 10.1006/meth.2001.1262

