# Supplementary material for: Prioritization of candidate genes in “QTL-hotspot” region for drought tolerance in chickpea (Cicer arietinum L.)
Source: Sci Rep. 2015 Oct 19;5:15296. doi: 10.1038/srep15296 (PMC4609953; doi:10.1038/srep15296)

**Prioritization of candidate genes in “*QTL-hotspot*” region for drought tolerance in chickpea (*Cicer arietinum* L.)**

Sandip M Kale^1+^, Deepa Jaganathan^1,2+^, Pradeep Ruperao^3,4^, Charles Chen^5^, Ramu Punna^6^, Himabindu Kudapa^1^, Mahendar Thudi^1^, Manish Roorkiwal^1^, Mohan AVSK Katta^1^, Dadakhalandar Doddamani^1^, Vanika Garg^1^, P B Kavi Kishor^2^, Pooran M Gaur^1^, Henry T Nguyen^7^, Jacqueline Batley^3^, David Edwards^3^, Tim Sutton^8,9^ and Rajeev K Varshney^1,3*^

^+^ These authors contributed equally to this work

^*^ Corresponding author: [r.k.varshney@cgiar.org](mailto:r.k.varshney@cgiar.org)

Tel: 0091 40 30713305; Fax: 0091 40 3071 3074/ 3075

**Supplementary Figure 1: Concordance between the genetic map and physical map (draft chickpea genome (CaGAv1.0)).**

The order of each bin on linkage and physical map was visualized using Strudel V. 1.12.03.20 software. Each green line represents the position of the bin marker on respective genetic and physical map. Straight lines indicate markers on genetic and physical maps are in same order while crossing of lines indicate interchange of those markers on genetic and physical maps. Excellent consistency in marker order was observed between genetic and physical map in case of Ca3, Ca4, Ca6 and Ca8 pseudomolecules whereas slight deviation was observed for other pseudomolecules (Ca1, Ca2, Ca5 and Ca7). This may be due to the misassembly in some regions in the draft genome of chickpea.

**Supplementary Figure 2: Marker trait association for 100 seed weight (100SDW) based on RMIP analysis.**

A Genomewide association study was carried out using SNPs from pseudomolecule 4 (Ca4) and phenotypic data of all the 19 traits. A RMIP (re-sampling model inclusion probability) score of ≥ 5 was used to identify significant associations. The grey coloured lines indicate the total number of MTAs identified for 100SDW trait on Ca4 while red coloured lines represent significant MTAs identified after RMIP analysis.


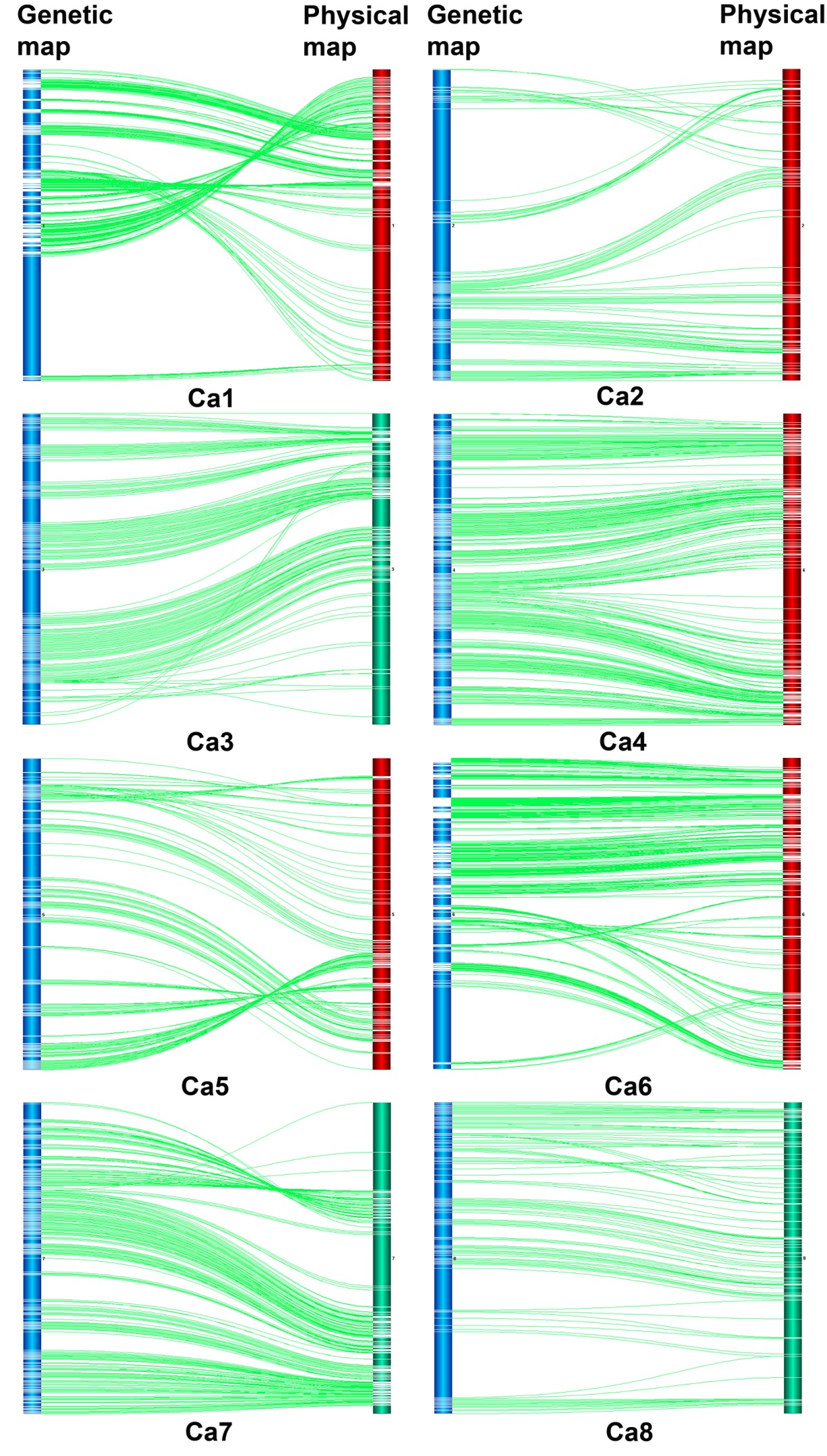


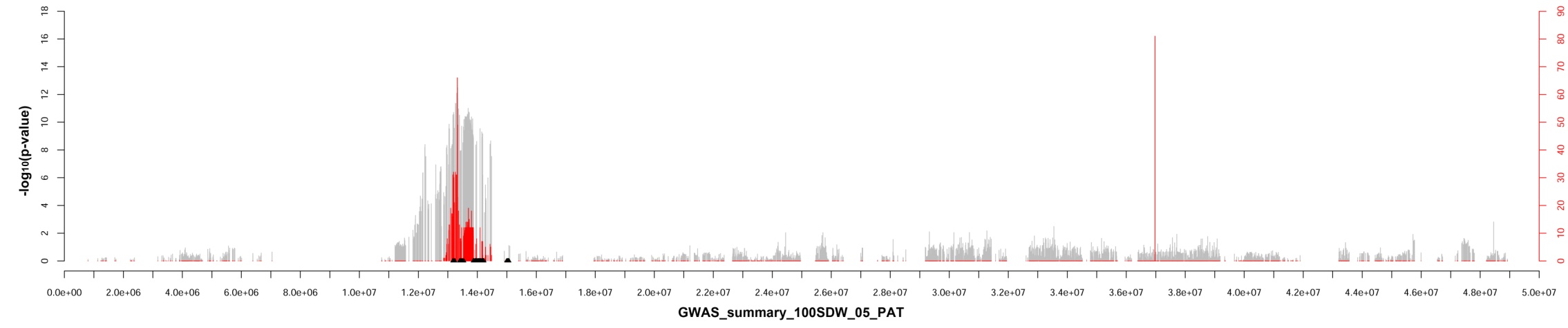

Supplement: Supplementary Figures 1-2 [file srep15296-s2.doc]
